# Therapeutic effect of human umbilical cord-derived mesenchymal stem cells on injured rat endometrium during its chronic phase

**DOI:** 10.1186/s13287-018-0777-5

**Published:** 2018-02-13

**Authors:** Lu Zhang, Ying Li, Chun-Yi Guan, Shi Tian, Xiao-Dan Lv, Jian-Hui Li, Xu Ma, Hong-Fei Xia

**Affiliations:** 1 Reproductive and Genetic Center of National Research Institute for Family Planning, Beijing, 100081 China; 2 Graduate School, Peking Union Medical College, Beijing, China; 3 Haidian Maternal & Child Health Hospital, Beijing, China

**Keywords:** UC-MSCs transplantation, Injured rat endometrium, Chronic phase, Therapeutic effect and mechanism

## Abstract

**Background:**

Repair deficiency after endometrial injury is an important reason for intra-uterine adhesions, amenorrhea, and infertility in females. Bone marrow-derived mesenchymal stem cell (BMSC) transplantation is effective in repairing the damaged endometrium. However, the possibility of using umbilical cord-derived MSCs (UC-MSCs) to treat endometrial injury is rarely reported.

**Methods:**

Ethanol (95%) was injected into rat uterus to establish a model of endometrial injury. UC-MSCs were injected through the tail vein, either as a single, twice, or thrice administration. Functional restoration of the uterus was assessed by testing embryo implantation rates. Endometrial morphological alteration was observed by hematoxylin and eosin staining. Endometrial fibrosis, markers of epithelial and stromal cells of endometrium, cell proliferation and angiogenesis, and inflammatory factors were detected using immunohistochemistry, Western blotting, and quantitative reverse-transcription polymerase chain reaction.

**Results:**

Endometrial morphology and embryo implantation rates were significantly improved on day 8 of transplantation among single-, twice-, or thrice-administered rats. Moreover, UC-MSCs could alleviate fibrosis in general, and reduced the expression of fibrosis markers, α-smooth muscle actin (α-SMA) and transforming growth factor (TGF)-β. The cell proliferation marker Ki-67 had a positive expression in the injured endometrium after UC-MSC transplantation. The endometrial stromal marker vimentin and epithelial marker cytokeratin-19 (CK-19) expressions were visibly increased. The expression of vascular markers CD31, vascular endothelial growth factor (VEGF)A, and matrix metalloprotein (MMP)9 was generally upregulated. Proinflammatory factors interferon (IFN)-γ, tumor necrosis factor (TNF)-α, and interleukin (IL)-2 were significantly downregulated in the rats administered UC-MSCs twice and thrice.

**Conclusions:**

UC-MSC transplantation contributed to the repair of endometrial injury and restoration of fertility, likely through the suppression of excessive fibrosis and inflammation, and enhancement of endometrial cell proliferation and vascular remodeling.

**Electronic supplementary material:**

The online version of this article (10.1186/s13287-018-0777-5) contains supplementary material, which is available to authorized users.

## Background

Endometrial health is one of the main factors in pregnancy. Serious injuries to the endometrium in women of childbearing age can often cause intra-uterine adhesion (IUA) or thin endometrium leading to amenorrhea, infertility, miscarriage, or other symptoms [1–3]. Considering the irreplaceable role of the uterus in the establishment and maintenance of pregnancy, it is of great clinical significance to study the structure and function of endometrial injury and explore the possible interventions to improve the uterine environment, promote the recovery of menstruation, and enhance the pregnancy rate [4, 5]. However, the complex nature of the uterine environment and function limits the possibilities of effective treatment. Therefore, it is an urgent gynecological need to explore novel treatment methods to repair endometrial damage, improve its microenvironment and tolerance status, and enhance the pregnancy rate in infertile patients.

In recent years, mesenchymal stem cells (MSCs) have been widely used in the treatment of various tissue and organ damage [6–8]. Exploring the potential of MSCs to repair or regenerate damaged endometrium has become a leading research topic. Wang et al. [9] have shown that bone marrow-derived MSC (BMSC) transplantation is effective in repairing damaged endometrium, likely through upregulating the estrogen receptor (ER) and progesterone receptor (PR) expressions. Jing et al. [10] and Zhao et al. [11] reported that BMSCs improved endometrial thickness, probably via their migratory and immunomodulatory properties. However, the differentiability of BMSCs decreases with an increase in the donor’s age, thereby limiting its further application. Human umbilical cord-derived stem cells (UC-MSCs), with multidirectional differentiation, a shorter proliferation time, low immunogenicity, easy extraction, long survival time after transplantation, and other characteristics, have become the preferred seed cells for transplantation [12, 13]. In vivo experiments confirmed that human UC-MSCs can repair vascular and tissue epithelial damage [14]. However, limited knowledge is available about the potential of human UC-MSCs to repair or regenerate damaged endometrium.

In the present study, human UC-MSCs were administered to rats via the tail vein to observe their effect on morphological reconstruction and functional regeneration of the damaged endometrium in rats and to explore the possible regeneration mechanism. Human UC-MSC transplantation may provide a novel tool to treat the currently intractable problem of injured endometrium.

## Methods

### Establishment of the rat endometrial injury model by 95% ethanol

Six- to eight-week-old male and female Sprague-Dawley (SD) rats (SPF level, Beijing Hua Fu Kang Biotechnology Co., Ltd., SCXK (Beijing) 2014-0004) were used in this study. They were housed four to five rats per cage at room temperature (23–25 °C), with humidity 40% to 60%, light/dark cycle 14 h/10 h, and free access to food and water.

The rats in the injured group received 350 mg/kg of 10% chloral hydrate anesthesia and body temperatures were maintained at 37 ± 0.5 °C. Under sterile surgical conditions, the abdominal wall was opened, the "Y" type uterus slowly picked out, and 0.3 mL 95% ethanol was injected; this was then rinsed with saline twice, and the wound was covered with wet gauze. The normal group did not undergo any treatment, and the sham group had physiological saline injected instead of 95% ethanol.

### Group and treatment

**Fig. 1:**
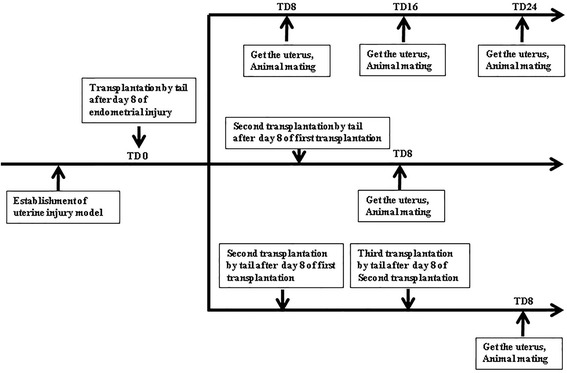
Experimental grouping schedule of the single, twice, and thrice transplantation groups. TD transplantation day

As shown in Fig. 1, the experiment was divided into a single transplantation group, a twice transplantation group, and a thrice transplantation group. Ninety-six SD rats were randomly assigned to four subgroups within the single transplantation group, including a group that did not undergo any treatment (normal group), a sham operation group that was injected with saline (sham group), an endometrial injury group (model group), and an MSC injected post-injury group (MSC group). Similarly, 32 SD rats were randomly assigned to four subgroups in the twice and thrice transplantation groups. For the sham group, 300 μL of saline was injected on each side of the Y-shaped uterus and then aspirated out. For the model group, 300 μL of 95% ethanol was injected on each side of the Y-shaped uterus and then aspirated out by saline. For the UC-MSC group, 1 × 10^7^/kg UC-MSCs were transplanted into each uterus post-injury. In the single transplantation group, the stem cells were injected into the tail vein on day 8 after injury, and then uteri were removed on days 8, 16, and 24 of transplantation (TD8, TD16, and TD24; Fig. 1), respectively, while some rats were subjected to mating experiments for fertility testing. In the twice and thrice transplantation groups, stem cells were injected into the tail vein on days 8 and 16, or days 8, 16, and 24 after endometrial injury, respectively, followed by uterine removal on TD8 and mating experiments as above.

### Isolation and characterization of MSCs

Human umbilical cords were obtained from full-term cesarean section surgery in Haidian Maternal and Child Health Hospital (Beijing, China). The patients had been informed a priori and had consented to donate. The MSCs were obtained by tissue mass culture. Briefly, under sterile conditions, umbilical cord tissue was cut open, the surface membrane gently peeled, the umbilical vein and artery removed, blood cleared from the tissue, and finally rinsed in saline. The tissue was cut as small as possible and evenly tiled on the culture dishes, and placed in a 37 °C, 5% CO_2_ incubator for 2 h until the small tissue pieces fully fitted in the culture dish. Medium was added to the dishes and left for culture. About 7–10 days later, cell growth was observed, and when culture dishes were covered with cells they were gently shaken to break down and remove the tissue; single cells continued to culture and were passaged. F5 generation cells were used to detect the surface antigen with the Human MSC Analysis Kit (BD Biosciences Franklin Lakes, NJ). Fat induction and bone formation experiments were performed to verify the differentiation potential of the MSCs. Adipogenesis was induced by inducers (2 mM dexamethasone, 2 mg/L insulin, 0.5 mM 3-isobutyl-1-methylxanthine, 0.2 mM indomethacin) for 14 days and confirmed by Oil Red O staining to show intracellular lipid accumulation. Osteogenesis was induced by inducers (2 mM dexamethasone, 1 M sodium glycerol phosphate, 10 mM vitamin C) for 28 days and calcium deposition was shown by Alizarin Red staining.

### Fertility testing

Uterine function was assessed by testing their capacity to receive fertilized ova and retain embryos for pregnancy. Five rats were sacrificed from every subgroup of the single, twice, or thrice transplantation groups. The rats were mated at 1:1 ratio with sexually mature male rats, euthanized 9 days after the appearance of vaginal plugs, and each uterus was examined for the number of fetuses.

### Hematoxylin and eosin (H&E) staining

H&E staining was used to observe the tissue structure. The tissues underwent regular processes of paraffin embedding, sectioning (4 μm), and H&E staining. TE2000-U inverted phase contrast microscope (Nikon, Tokyo, Japan) was used to observe rat uterine histomorphological changes. The endometrial thickness and the number of glands were recorded on H&E slides. Five fields in each image were selected for counting.

### Masson trichrome staining

Masson trichrome staining was used to detect fibrosis. The tissues underwent regular paraffin embedding, sectioning (4 μm), and Masson staining. TE2000-U inverted phase contrast microscope (Nikon) was used to observe fibrosis in different groups. Five fields in each image were selected for counting. ImageJ software was applied to analyze the level of interstitial fibrosis in the endometrium.

### Histological analysis

The expression of vimentin, Ki-67, and CD31 proteins was detected by immunohistochemistry. Slides were incubated with mouse anti-vimentin monoclonal antibody (Santa Cruz, CA, USA; 1:100), mouse anti-Ki-67 monoclonal antibody (Abcam, Cambridge, UK; 1:200), and mouse anti-CD31 monoclonal antibody (Santa Cruz; 1:100), respectively, followed by further incubation with horseradish peroxidase (HRP)-conjugated goat anti-mouse IgG (ZSJQ-Bio, Beijing, China, 1:100). The antibody stains were developed by the addition of diaminobenzidine (DAB) and the nucleus was stained with hematoxylin. Anti-human nuclear antibody MAB1281 (Millipore, Billerica, MA, USA) and TRITC-conjugated goat anti-mouse IgG (ZSJQ-Bio; 1:100) were used to detect the migration of the transplanted human UC-MSCs to the injured uterus by immunofluorescence; the nucleus was stained with DAPI.

### Western blotting

Equal amounts of proteins extracted from the different groups were run on 10% SDS-PAGE and transferred to PVDF membranes (Amersham, St. Albans, UK). Membranes were incubated with mouse anti-vimentin monoclonal antibody (Santa Cruz; 1:500) and mouse anti-CK-19 monoclonal antibody (Santa Cruz; 1:500) respectively overnight at 4 °C. The membranes were then incubated with HRP-conjugated goat anti-mouse IgG (ZSJQ-Bio; 1:2000) at room temperature for 1 h. ECL detection reagents (Millipore) were added to the membranes and they were immediately exposed to x-ray film (Kodak, Rochester, NY, USA).The β-actin signal was used as a control to ensure equal protein loading.

### Quantitative reverse-transcription polymerase chain reaction (qRT-PCR)

Total RNA from rat uteri was extracted using Trizol (Invitrogen, Carlsbad, CA, USA) according to the manufacturer’s protocols; 1 μg of total RNA was subjected to reverse transcription of mRNA using oligo dT as a primer and a reverse transcription kit (Transgen Biotech, Beijing, China) to generate total cDNA. The quantitative PCR was then carried using primers shown in Table 1 and FastStart Universal SYBR Green Master (Toyobo, Osaka, Japan) with the 7500 Real-Time PCR System (Applied Biosystems, Foster City, CA, USA). GAPDH was used for normalization. Each sample in each group was detected in triplicate. The experiment was repeated at least three times.

**Table 1:** Primer sequences

Gene name	Primer sequence
rat-α-SMA-qPCR-F	CCTCTTCCAGCCATCTTTCAT
rat-α-SMA-qPCR-R	CGAGAGGACGTTGTTAGCATAG
rat-TGF-β1-qPCR-F	AGAGCCCTGGATACCAACTA
rat-TGF-β1-qPCR-R	CAACCCAGGTCCTTCCTAAAG
rat-Ki-67-qPCR-F	CTGCAGAGAAGGTTGGGATAAA
rat-Ki-67-qPCR-R	CTGACTTTGCCCAGAGATGAA
rat-VEGFA-qPCR-F	GAAGACACAGTGGTGGAAGAAG
rat-VEGFA-qPCR-R	ACAAGGTCCTCCTGAGCTATAC
rat-MMP9-qPCR-F	GCTGCTCCAACTGCTGTATAA
rat-MMP9-qPCR-R	TGGTGTCCTCCGATGTAAGA
rat-CD31-qPCR-F	GCCGTCAAATACTGGGTTAGT
rat-CD31-qPCR-R	GCACTGTACACCTCCAAAGAT
rat-IFN-γ-qPCR-F	GTGAACAACCCACAGATCCA
rat-IFN-γ-qPCR-R	GAATCAGCACCGACTCCTTT
rat-TNF-α-qPCR-F	CCCAATCTGTGTCCTTCTAACT
rat-TNF-α-qPCR-R	CAGCGTCTCGTGTGTTTCT
rat-IL-2-qPCR-F	GCAGGCCACAGAATTGAAAC
rat-IL-2-qPCR-R	CCAGCGTCTTCCAAGTGAA
rat-IL4-qPCR-F	GGTGAACTGAGGAAACTCTGTAG
rat-IL4-qPCR-R	TCCAGGAAGTCTTTCAGTGTTG
rat-IL-10-qPCR-F	AGTGGAGCAGGTGAAGAATG
rat-IL-10-qPCR-R	GAGTGTCACGTAGGCTTCTATG
rat-GAPDH-qPCR-F	GCAAGGATACTGAGAGCAAGAG
rat-GAPDH-qPCR-R	GGATGGAATTGTGAGGGAGATG

### Statistical analysis

Statistical analysis was performed with SPSS version 20.0. Quantitative data are expressed as mean ± standard deviation from at least three independent experiments. Two-group comparisons were performed using a *t* test and multiple-group comparisons were determined by one-way analysis of variance (ANOVA). *P* < 0.05 was considered statistically significant.

## Results

### UC-MSCs express specific surface antigens, possess multilineage differentiation potential, and demonstrate homing tendency to injury site

The expression of UC-MSC surface antigen and differentiation potential were examined by human MSC analysis kit. In the cells, separated from human umbilical cord, the expression rate of CD73 was 99.89% while that of CD105 was 96.88%, CD90 98.46%, and CD44 99.60%, all of which was higher than 95%. The expression of CD34, CD11b, CD19, CD45, and HLA-DR was lower than 5% (Additional file 1: Figure S1B–G). In addition, after induction in vitro, these cells exhibited the ability to differentiate into adipocytes and osteoblasts (Additional file 1: Figure S1H–K). Taken together, the cells matched the criteria defined by the International Society for Cellular Therapy (ISCT) position paper [15] and were defined as UC-MSCs. In addition, UC-MSCs injected into tail vein were found to accumulate in the damaged endomembrane area (Additional file 1: Figure S1K).

### UC-MSCs ameliorate the macroscopic appearance and morphological features of the uterus

**Fig. 2:**
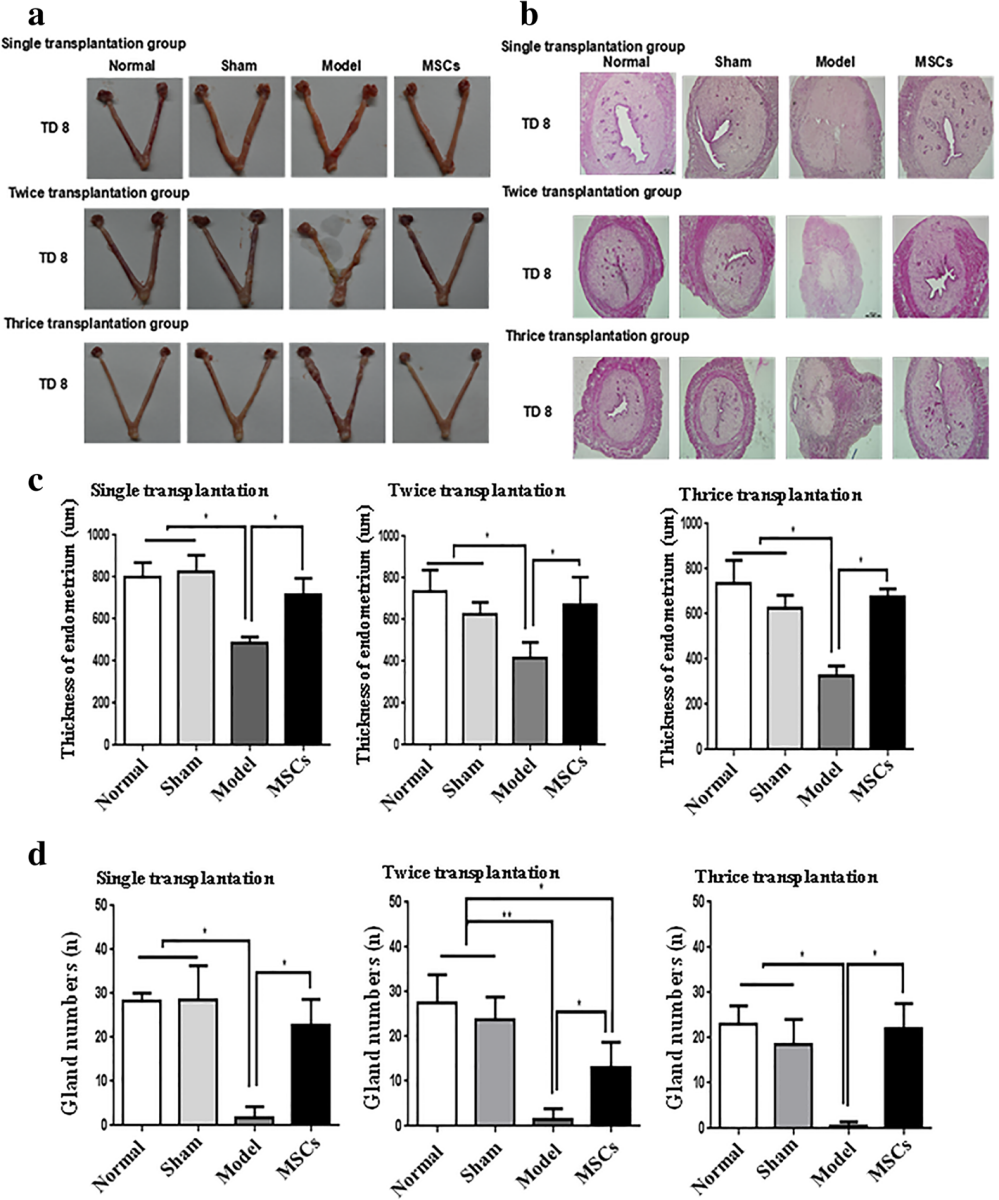
Uterine morphological features and changes. **a** The uterus specimen. **b** H&E staining of rat uterine tissue (50×). **c** The endometrial thickness at transplantation day 8 (TD8) in single, twice, and thrice transplantation groups. **d** The gland numbers at TD8 in single, twice, and thrice transplantation groups. *P < 0.05, **P < 0.01. MSC mesenchymal stem cell

The macroscopic appearance of the uterus in the normal and sham groups was smoother and tougher than in the model group. After single or multiple transplantations of UC-MSCs, the macroscopic appearance of the uterus remained like that of a normal uterus (Fig. 2a). From H&E stained images, the endometrial structure of the normal group seemed more complete, epithelial cells were arranged closely, and blood vessels and glands were clearly visible; the basic endometrial structure of the sham group did not show any change. In the model group, the endometrium was poor and IUA or endometrial thinning was serious. The uterus recovered well in the MSC transplantation group, and the glands and blood vessels were obvious. The effects of single transplantation were quite good at TD8, but deteriorated with time (Additional file 2: Figure S2); the multiple transplantation showed a better result at TD8 (Fig. 2b).

The endometrial thickness and the number of endometrial glands were not significantly different in the normal group compared with the sham group, but were significantly reduced at TD8, TD16, and TD24 in the single transplantation group and at TD8 in the multiple transplantation groups (*P* < 0.05; Figs. 2c, d and Additional file 2: Figure S2C, D). In the single transplantation group, the endometrial thickness and the number of endometrial glands almost returned to normal levels at TD8, but the repair effect was weaker at TD16 and TD24. The repair effect of multiple injections at TD8 was similar to that of single transplantation at TD8.

### UC-MSC transplantation improves fertility in rats

**Fig. 3:**
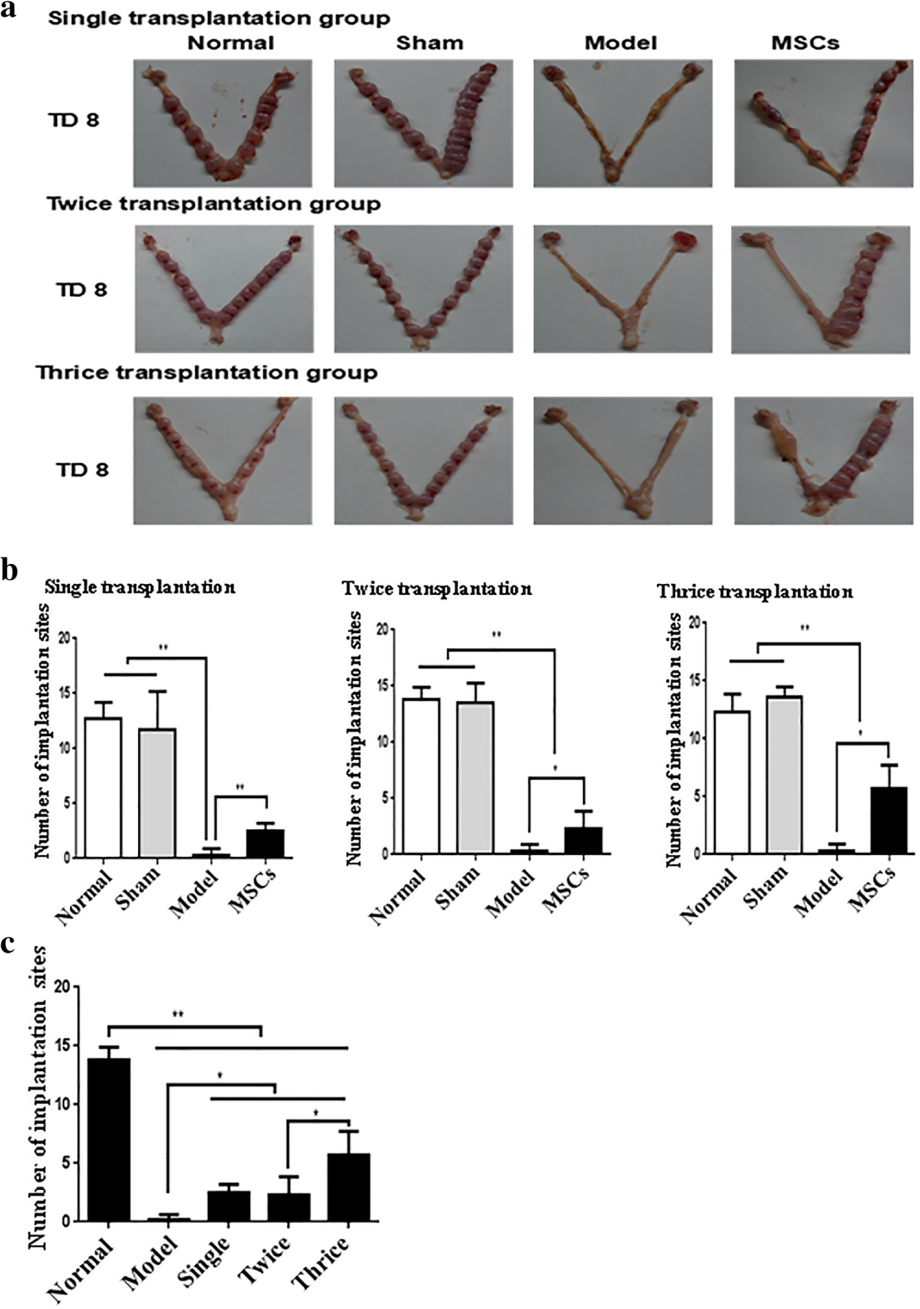
Human UC-MSC transplantation restores receptive fertility of injured uterus. **a** Embryos implanted in the uterus in single, twice, and thrice transplantation groups. **b** The affected rat pregnancy number by human UC-MSC transplantation in single, twice, and thrice transplantation groups. *P < 0.05, **P < 0.01. MSC mesenchymal stem cell, TD8 transplantation day 8

IUA or a thin endometrium is closely related to embryonic implantation; endometrial damage can lead to embryo-implantation failure or a reduced embryo-implantation rate. In order to detect the effect of MSCs on the repair of the endometrium, we looked at the embryo-implantation efficiency in the different groups of rats. As shown in Fig. 3a and Additional file 3: Figure S3A, compared with the model group the embryo-implantation efficiency was significantly increased at TD8 (*P* < 0.01) and TD16 (*P* < 0.05), though not significantly at TD24, in the single transplantation group. In the multiple transplantation groups, the number of implanted embryos was remarkably raised at TD8 (*P* < 0.05). The implantation numbers were higher in the thrice transplantation group than in the single transplantation group (*P* < 0.05; Fig. 3b). These results suggest that UC-MSC transplantation can repair injured endometrium and partially restore fertility, with the thrice transplantation group showing the best repair effect (Fig. 3b, c).

### UC-MSC transplantation relieves endometrial fibrosis

**Fig. 4:**
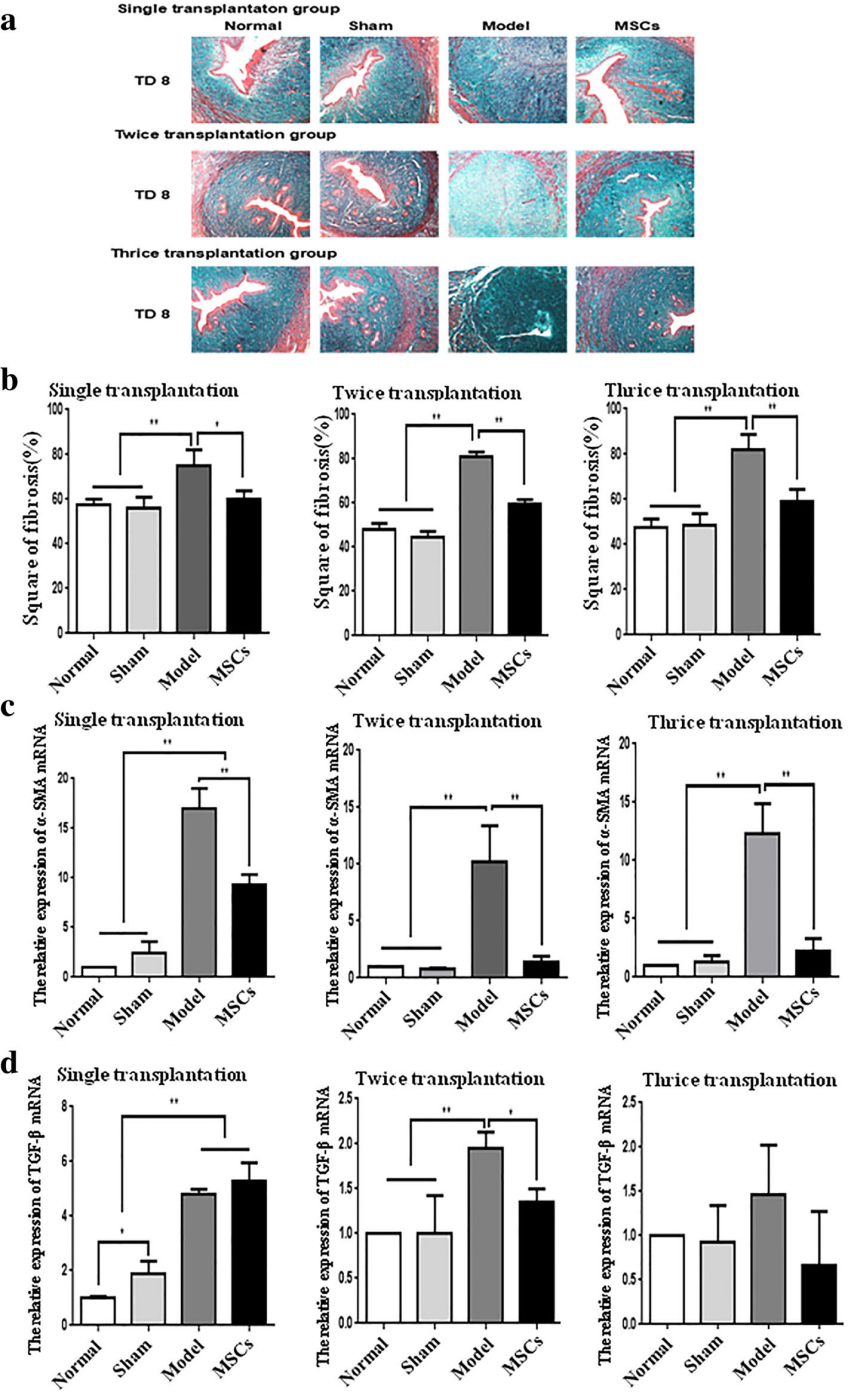
Human UC-MSC transplantation relieves endometrial fibrosis. **a** Masson’s trichrome staining of uterine scars (100×). **b** The fibrosis in single, twice, and thrice transplantation groups. **c** Alpha smooth muscle actin (α-SMA) mRNA expression in single, twice, and thrice transplantation groups was detected by qRT-PCR. **d** Transforming growth factor (TGF)-β mRNA expression in single, twice and thrice transplantation groups was detected by qRT-PCR. GAPDH serves as an internal reference for qRT-PCR. *P < 0.05, **P < 0.01. MSC mesenchymal stem cell, TD8 transplantation day 8

When the uterine tissue injury is serious, interstitial fibrous connective tissue proliferates and repairs the damaged tissue, finally leading to fibrotic lesions. Therefore, the extent of fibrosis of the endometrium can directly reflect the level of endometrial damage and the effect of stem cell therapy. Masson's trichrome staining showed a significant difference between the normal, sham, and injured uterine tissues. The injured uterine cavity was severely adhered compared to normal endometrium and the myometrium was replaced by collagen; the MSC-treated group had a better repair effect compared to the model group (Fig. 4a). As shown in Fig. 4b and Additional file 4: Figure S4B, all rats in the model group had serious fibrosis; UC-MSC injection significantly attenuated endometrial fibrosis at TD8 in the single (*P* < 0.05), twice (*P* < 0.01), and thrice transplantation groups (*P* < 0.01; Fig. 4b) and TD16 of the single transplantation group (P < 0.01), but was virtually ineffective at TD24 in the single transplantation group (Additional file 4: Figure S4B).

To detect the degree of fibrosis in the endometrium, we examined the expression of the fibrosis markers α-smooth muscle actin (SMA) and transforming growth factor (TGF)-β by qRT-PCR (Fig. 4c, d and Additional file 4: Figure S4C, D). UC-MSC treatment significantly inhibited the α-SMA upregulation induced by endometrial injury at TD8 in the single (*P* < 0.01), twice (*P* < 0.01), or thrice transplantation (*P* < 0.01; Fig. 4c) groups, and TD16 in the single transplantation group (*P* < 0.01; Additional file 4: Figure S4C), but had no significant effect at TD24 in the single transplantation group (Additional file 4: Figure S4C). The endometrial injury-induced TGF-β upregulation was evidently suppressed by twice transplantation (*P* < 0.05; Fig. 4d). All these results show that UC-MSC transplantation can partially alleviate the degree of fibrosis, but the multiple transplantation groups had a better repair effect compared to the single transplantation group.

### UC-MSC transplantation promotes the proliferation of endometrial cells

**Fig. 5:**
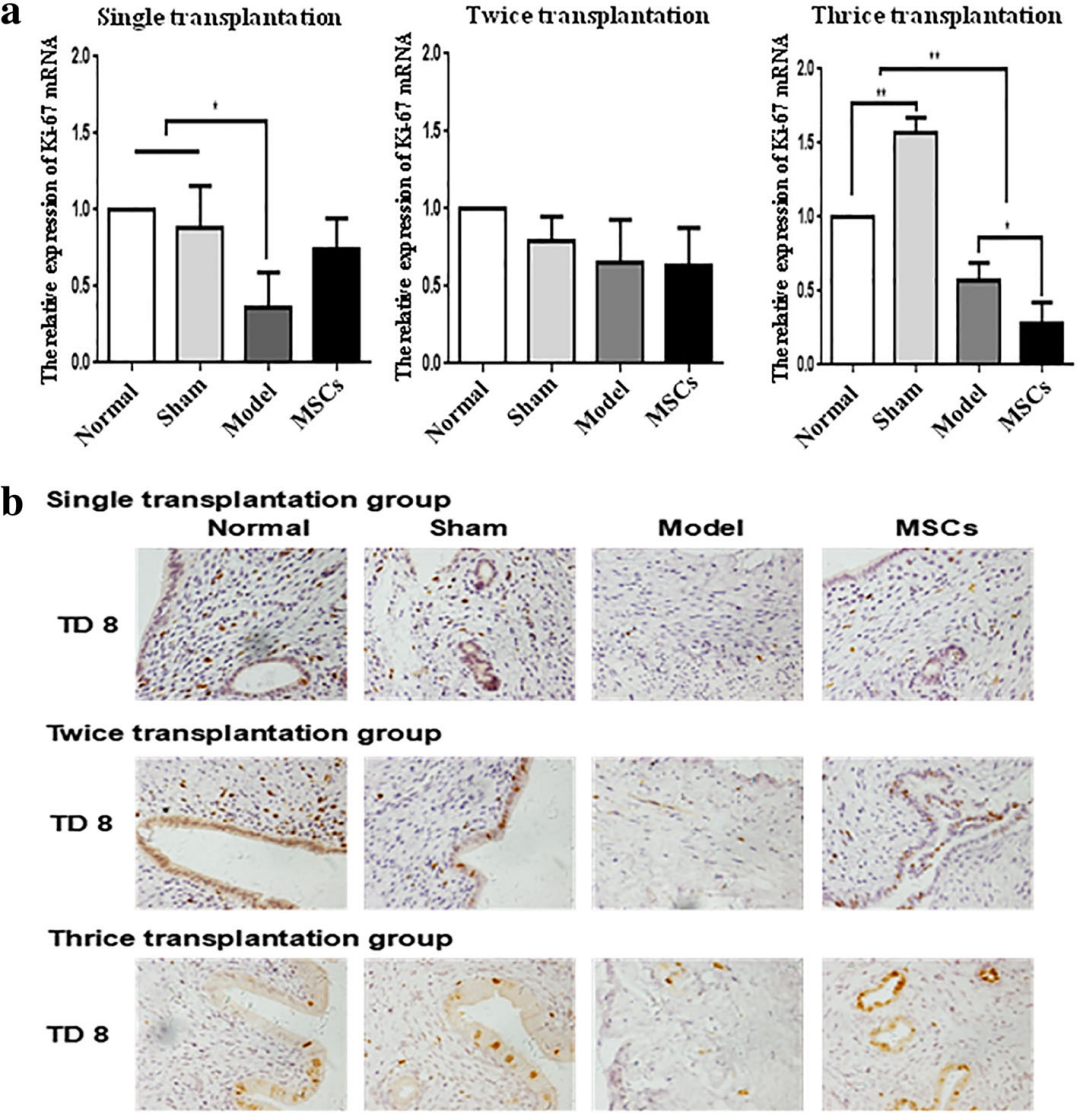
Human UC-MSC transplantation promotes endothelial cell proliferation. **a** Ki-67 protein expression in single, twice, and thrice transplantation groups was detected by immunohistochemistry (400×). **b** Ki-67 mRNA expression in single, twice, and thrice transplantation groups was detected by qRT-PCR. GAPDH serves as an internal reference for qRT-PCR. *P < 0.05, **P < 0.01. MSC mesenchymal stem cell, TD8 transplantation day 8

Ki67 is a kind of nuclear antigen whose function is closely related to mitosis and is indispensable in cell proliferation [16]. The expression of Ki67 is also an important index to study the effect of endometrial repair. qRT-PCR was used to detect the Ki-67 mRNA level (Fig. 5a and Additional file 5: Figure S5A). All model groups had lower Ki67 levels compared with those in the other groups. Ki67 was enhanced at TD16 and TD24 in the single transplantation group (*P* < 0.05). Immunohistochemical results showed that positive Ki67 signals were mainly located in the glandular and luminal epithelium. The positive staining of Ki67 was weak in the model group compared with that in the normal and sham group. The positive signals of Ki67 among single or multiple transplantation groups were stronger than in the model group (Fig. 5b and Additional file 5: Figure S5B). The repair effect was not evident at TD8 in single, twice, or thrice administration groups.

### UC-MSC transplantation promotes the regeneration of endometrial cells

**Fig. 6:**
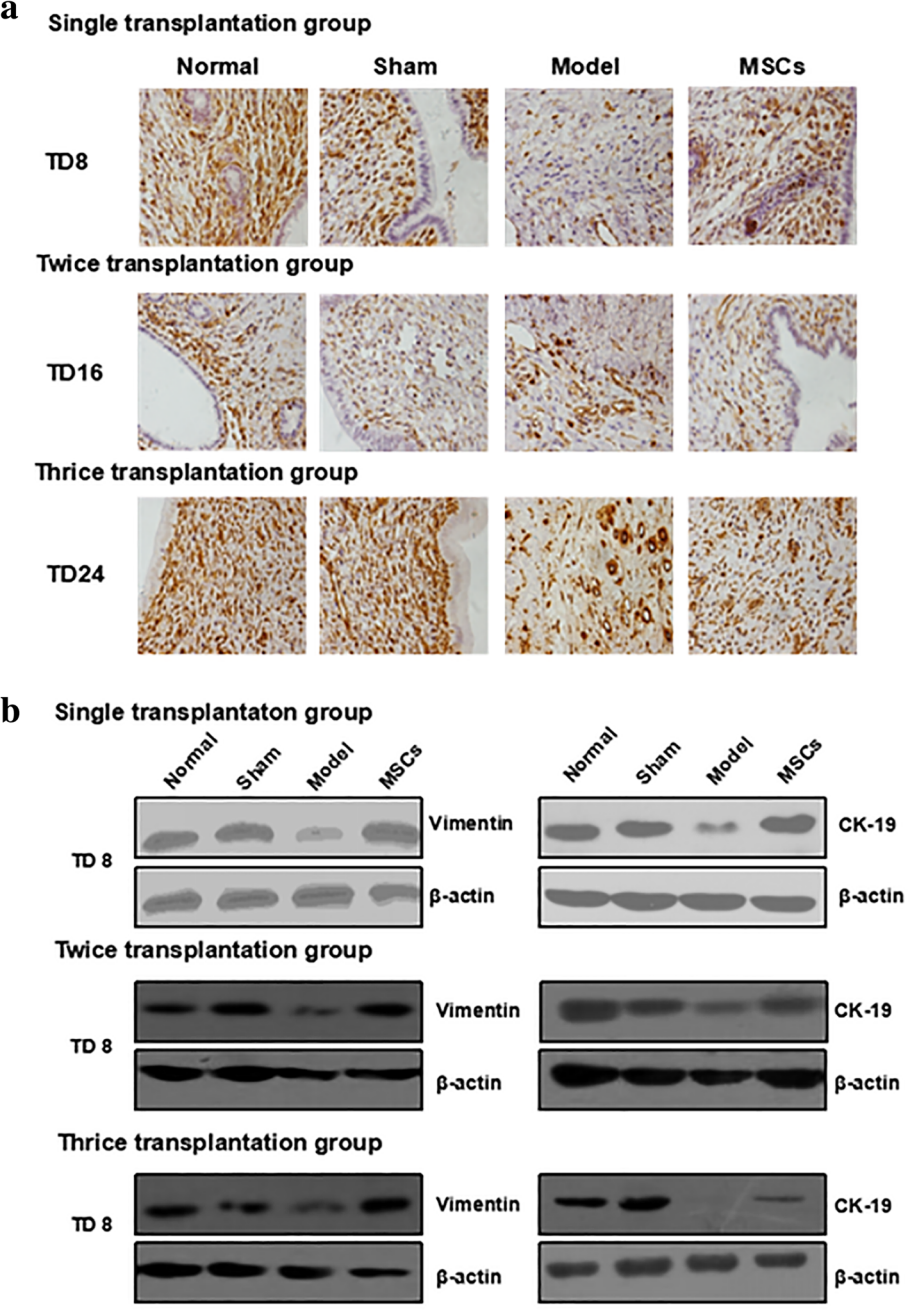
Human UC-MSC transplantation promotes the regeneration of endometrial cells. **a** Vimentin protein expression in single, twice, and thrice transplantation groups was detected by immunohistochemistry (400×). **b** Vimentin protein and cytokeratin (CK)-19 protein expression in single, twice and thrice transplantation groups was detected by Western blot. MSC mesenchymal stem cell, TD8 transplantation day 8

Vimentin is one of the proteins of the middle filament responsible for maintaining integrity of the cytoskeleton, and represents uterine stromal cells. Cytokeratin-19 (CK-19), mainly distributed in epithelial cells, is the main skeletal protein in keratinocytes. CK-19 can maintain the integrity and continuity of epithelial tissue and is closely related to endometrial and glandular epithelial cell proliferation and differentiation. Expression of vimentin and CK-19 also gives us an important index to study the degree of endometrial injury and repair. In this study, immunohistochemistry detected the localization of vimentin (Fig. 6a and Additional file 6: Figure S6A). The results showed that the positive signals of vimentin were observed in uterine stromal cells. Vimentin staining was weaker in the model group than in the normal group, and reached near-normal levels after UC-MSC transplantation in the single, twice, or thrice administration groups. The expression of vimentin and CK-19 in the rat uterus was analyzed by Western blotting (Fig. 6b and Additional file 6: Figure S6B). Compared with the normal and sham groups, the protein levels of vimentin and CK-19 were significantly decreased in the model group. After UC-MSC treatment, the vimentin level was evidently increased to near-normal levels in single, twice, or thrice administration groups. CK-19 protein was raised at TD8 in all three transplantation groups, but not at TD16 and TD24 in the single transplantation group. These results suggest that UC-MSC transplantation promotes the regeneration of endometrial cells.

### UC-MSC transplantation promotes angiogenesis

**Fig. 7:**
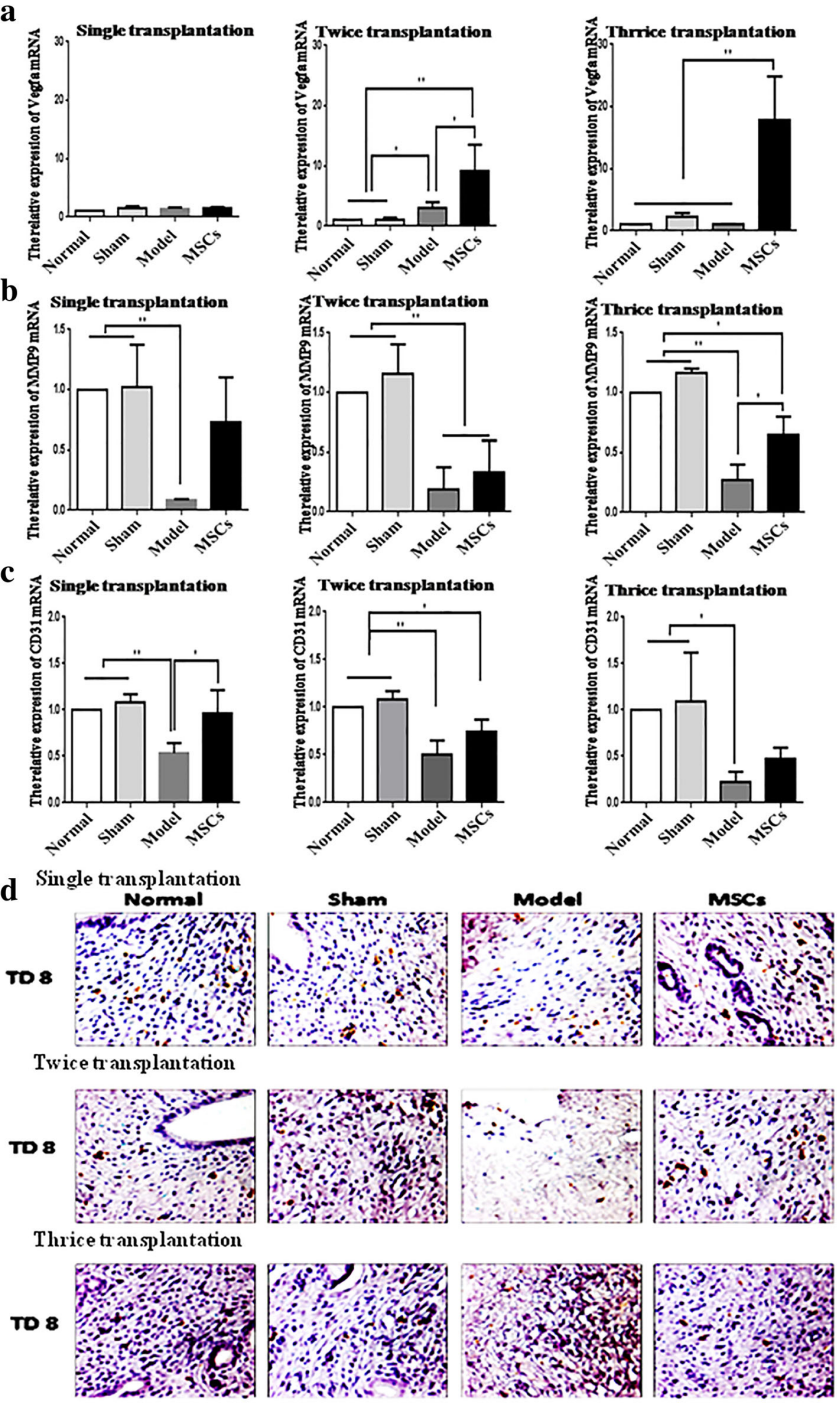
UC-MSC transplantation promotes angiogenesis. **a** Vascular endothelial growth factor (VEGF)A mRNA expression in single, twice, and thrice transplantation groups was detected by qRT-PCR. **b** Matrix metalloprotein (MMP)9 mRNA expression in single, twice, and thrice transplantation groups was detected by qRT-PCR. **c** CD31 mRNA expression in single, twice, and thrice transplantation groups was detected by qRT-PCR. GAPDH serves as an internal reference for qRT-PCR. *P < 0.05, **P < 0.01. **d** CD31 protein expression in single, twice, and thrice transplantation groups was detected by immunohistochemistry (400×). MSC mesenchymal stem cell, TD8 transplantation day 8

Vascular endothelial growth factor (VEGF) is vital for the formation of new vessels and re-epithelialization of the endometrium [17]. Studies have reported a positive feedback between matrix metalloprotein (MMP)9 and VEGF in angiogenesis [18–20]. CD31 is mainly used to detect the presence of endothelial cell tissue for the assessment of angiogenesis [21, 22]. Hence, the expression of CD31, VEGF, and MMP9 can exhibit angiogenesis of injured endometrium. The expression of VEGFA, MMP9, and CD31 in rat uterine tissue was detected by qRT-PCR (Fig. 7a–c and Additional file 7: Figure S7A–C). VEGFA expression was higher in the multiple transplantation groups than in the model group. MMP9 and CD31 mRNA expressions were significantly decreased by endometrial injury. The downregulation of MMP9 expression was restrained in the thrice transplantation group (*P* < 0.05) while that of CD31 was restored at TD8 in the single transplantation group.

To evaluate the new vessel formation directly, immunohistochemistry was used to detect the location of CD31 in the uterus (Fig. 7d and Additional file 7: Figure S7D). The positive signals of CD31 were observed in the stromal cells; CD31 stain was lighter in the model group than in the normal and sham groups, but was intense in UC-MSC-treated group compared with the model group. These results show that UC-MSCs can promote the formation of blood vessels.

### UC-MSCs regulate the release of inflammatory factors

**Fig. 8:**
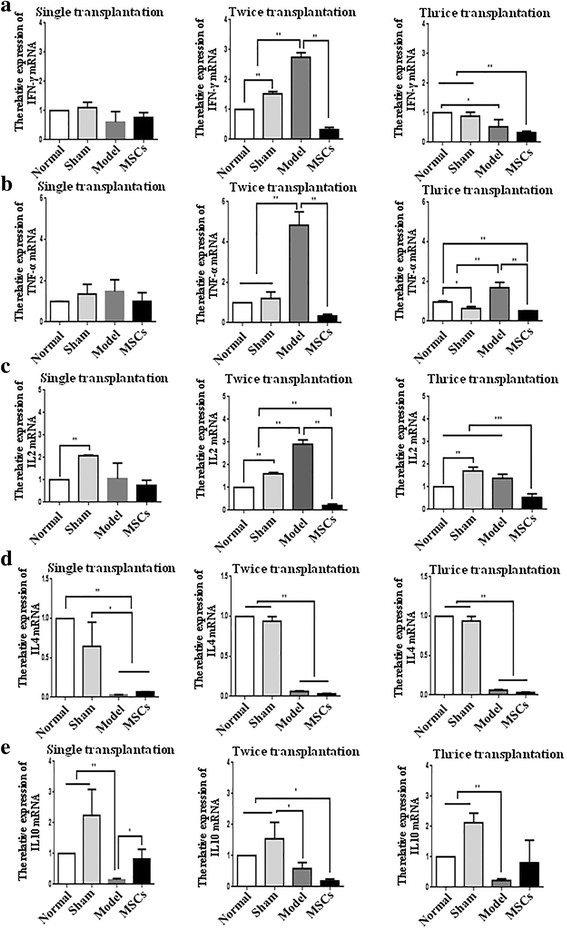
Human UC-MSCs regulate the release of inflammatory factors. **a** Interferon (IFN)-γ mRNA expression in single, twice, and thrice transplantation groups was detected by qRT-PCR. **b** Tumor necrosis factor (TNF)-α mRNA expression in single, twice, and thrice transplantation groups was detected by qRT-PCR. **c** Interleukin (IL)-2 mRNA expression in single, twice, and thrice transplantation groups was detected by qRT-PCR. **d** IL-4 mRNA expression in single, twice, and thrice transplantation groups was detected by qRT-PCR. **e** IL-10 mRNA expression in single, twice, and thrice transplantation groups was detected by qRT-PCR. GAPDH serves as an internal reference for qRT-PCR. *P < 0.05, **P < 0.01. MSC mesenchymal stem cell, TD8 transplantation day 8

MSCs migrate to the injured site to produce a large number of proinflammatory factors, anti-inflammatory cytokines, and growth factors [23–25]. Here, we detected the expression of the proinflammatory factors interferon (IFN)-γ, tumor necrosis factor (TNF)-α, and interleukin (IL)-2, and the anti-inflammatory cytokines IL4 and IL-10 by qRT-PCR (Fig. 8). The expression of the proinflammatory factors IFN-γ, TNF-α, and IL-2 in the model group was higher than that at TD8 in the twice and thrice transplantation groups, but not significantly different to TD8 in the single transplantation group. The expression of IL-4 showed no significant difference between the model group and the UC-MSC-treated groups. Compared with the model group, IL-10 expression was noticeably enhanced in the uterus of the single transplantation group at TD8 (*P* < 0.05).

## Discussion

In this study, we used human UC-MSCs to treat endometrial injury by single and multiple transplantations and found that human UC-MSCs can repair the injured rat endometrium, thereby improving fertility in rats.

We chose human UC-MSCs for repairing endometrial injury for the following reasons. Several kinds of MSCs are used in scientific and clinical research. For instance, human UC-MSCs, BMSCs, or adipose tissue-derived MSCs (A-MSCs) are used to improve the function of many tissues [26–28]. Some studies have reported a comparison of the biological characteristics and roles of UC-MSCs with those of BMSCs or A-MSCs. Heo et al. investigated the immunophenotype, proliferative potential, multilineage differentiation, and immunomodulatory capacity of UC-MSCs, A-MSCs, and BMSCs, and revealed that BMSCs and A-MSCs represent the optimal stem-cell source for tissue engineering and regenerative medicine based on their tri-lineage differentiation potential and immunomodulatory effects [29]. Oloyo et al. contrasted the role of mesenchymal stromal/stem cells in tumor growth by assessing 183 original research articles comprising 338 experiments, and found that tumor growth was inhibited in 80% of the experiments that used UC-MSCs, whereas it was promoted in 64% and 57% of the experiments using BMSCs and A-MSCs, respectively [30]. In comparison to BMSCs, UC-MSCs have higher proliferative capacity, a higher rate of chondrogenic differentiation, and higher expression of CD146 [31]. Moreover, considering the known advantages of UC-MSCs such as accessibility, painless acquisition, and low immunogenicity, we chose human UC-MSCs to repair endometrial injury in our rats.

To ascertain that self-repair by the uterus did not happen, we designed the chronic phase, characterized by permanent injury, to be repaired by UC-MSCs after 8 days. Studies have confirmed that BMSCs can promote endometrial and muscle cell proliferation, promote microvascular regeneration, and recover the endometrial embryo-implantation ability [32]. This is consistent with our findings. Our study shows that endometrial repair is good at TD8 in the single transplantation group, but this effect diminishes over time; hence, we chose to increase the number of transplantations to test the repair capacity after multiple transplantations of MSCs.

The repair of the endometrium, similar to most trauma repair processes, generally involves three phases of inflammation, tissue repair, and tissue remodeling [5]. After endometrial injury, endometrial epithelial cell defects, lack of endometrial coverage of the uterine cavity, and interstitial cell hyperplasia lead to endometrial fibrosis, adhesion, and inhibition of proliferation [33]. Our experimental results showed that UC-MSC transplantation can inhibit the occurrence of fibrosis, downregulate the expression of α-SMA and TGF-β, and improve the endometrial cell viability. The endometrium consists of luminal and glandular epithelia, stromal cells, and vessels [34]. The endometrial stromal marker vimentin and epithelial marker CK-19 expression was visibly increased, and the expression of vascular markers CD31, VEGFA, and MMP9 was generally upregulated. Proinflammatory factors IFN-γ, TNF-α, and IL-2 were significantly downregulated in twice and thrice transplantation groups. These results suggest that UC-MSCs can repair endometrial injury through relieving endometrial fibrosis and the inflammatory reaction, promoting proliferation and regeneration of endometrial cells and angiogenesis.

In this study, we found that multiple transplantations could more effectively improve rat fertility than a single transplantation in treating endometrial damage. We can justify this as follows. Firstly, the expression of MMP9 and VEGF was significantly increased in the multiple-dose administration, but there was no significant difference in the single-dose administration. MMP9 and VEGF are angiogenic factors implicated in the embryo invasion process [35]. MMP9-null mouse embryos exhibit deficiencies in trophoblast differentiation and invasion after implantation [36]. VEGF can significantly enhance endometrial epithelial cell adhesive properties and embryo outgrowth [37]. Secondly, the expression of the proinflammatory factors IFN-γ, TNF-α, and IL-2 was significantly decreased in the multiple-dose administration, but there was no significant difference in the single-dose administration. IFN-γ, TNF-α, and IL-2 belong to the T-helper (Th)1 type cytokines, and mediate pregnancy loss [38]. The upregulation of vascular markers and downregulation of proinflammatory factors may be the main reasons that the number of implanted embryos is higher in the groups with multiple UC-MSC transplantations than that in the single UC-MSC transplantation group. Although we saw some positive results in our study, there are still some ongoing questions; for example, would allogeneic UC-MSCs induce a potential allogeneic response, and would a second or third dose consolidate the initial effects observed.

## Conclusion

In this study, we found that human umbilical cord-derived mesenchymal stem cells can restore the endometrial thickness, glands, implanted embryos, alleviate excessive fibrosis caused by endometrial injury, promote blood vessel growth and endothelial cell proliferation, downregulate some proinflammatory factors, and upregulate some anti-inflammatory factors to restore uterine structure and function. With multiple transplantations of human UC-MSCs, some of the functions recovered better. Considering the significance of the endometrium in embryonic implantation and pregnancy, treatment of endometrial injury has a very important clinical significance and value.

## Additional files

Additional file 1: Figure S1. Identification of stem cells. (A–F) Flow cytometry analysis of immune-markers in human UC-MSCs. (G) Normal human UC-MSCs was stained by Oil Red O (200×). (H) Adipogenesis was confirmed by Oil Red O staining to show intracellular lipid accumulation (200×). (I) Normal human UC-MSCs were stained by Alizarin Red (200×). (J) Osteogenesis was confirmed by Alizarin Red staining to show calcium deposition (200×). (K) MAB1281 expression in normal group and MSCs transplantation group at TD8 (200×). (TIF 6471 kb)

Additional file 2: Figure S2. Uterine morphological features and changes of single transplantation group on TD16 and TD24. (A) A uterus specimen. (B) H&E staining of rat uterine tissue (50×). (C) The endometrial thickness at TD16 and TD24. (D) The gland numbers at TD16 and TD24. *P < 0.05. (TIF 5634 kb)

Additional file 3: Figure S3. Human UC-MSC transplantation restores receptive fertility of the single transplantation group at TD16 and TD24. (A) Embryos implanted in the uterus on TD16 and TD24. (B) The effect on rat pregnancy number by human UC-MSC transplantation at TD16 and TD24. *P < 0.05, **P < 0.01. (TIF 4634 kb)

Additional file 4: Figure S4. Human UC-MSC transplantation relieves endometrial fibrosis of the single transplantation group at TD16 and TD24. (A) Masson’s trichrome staining of the single transplantation group at TD16 and TD24 (100×). (B) The fibrosis at TD16 and TD24. (C) α-SMA mRNA expression at TD16 and TD24 was detected by qRT-PCR. (D) TGF-β mRNA expression at TD16 and TD24 was detected by qRT-PCR. GAPDH serves as an internal reference for qRT-PCR. *P < 0.05, **P < 0.01. (TIF 6938 kb)

Additional file 5: Figure S5. Human UC-MSC transplantation promotes endothelial cell proliferation in the single transplantation group at TD16 and TD24. (A) Ki-67 mRNA expression at TD16 and TD24 was detected by qRT-PCR. GAPDH serves as an internal reference for qRT-PCR. *P < 0.05, **P < 0.01. (B) Ki-67 protein expression at TD16 and TD24 was detected by immunohistochemistry (400×). (TIF 6638 kb)

Additional file 6: Figure S6. Human UC-MSC transplantation promotes endometrial cell regeneration of the single transplantation group at TD16 and TD24. (A) Vimentin protein expression at TD16 and TD24 was detected by immunohistochemistry (400×). (B) Vimentin protein and CK-19 protein expression at TD16 and TD24 was detected by Western blot. (TIF 8220 kb)

Additional file 7: Figure S7. UC-MSC transplantation promotes angiogenesis on TD16 and TD24 in the single transplantation group. (A) VEGFA mRNA expression at TD16 and TD24 was detected by qRT-PCR. (B) MMP9 mRNA expression at TD16 and 24 was detected by qRT-PCR. (C) CD31 mRNA expression at TD16 and TD24 was detected by qRT-PCR. GAPDH serves as an internal reference for qRT-PCR. *P < 0.05, **P < 0.01. (D) CD31 protein expression at TD16 and TD24 was detected by immunohistochemistry (400×). (TIF 8221 kb)

## Abbreviations

A-MSC: Adipose tissue-derived mesenchymal stem cell
BMSC: Bone marrow-derived mesenchymal stem cell
CK: Cytokeratin
H&E: Hematoxylin and eosin
IFN: Interferon
IL: Interleukin
ISCT: International Society for Cellular Therapy
IUA: Intra-uterine adhesion
MMP: Matrix metalloprotein
MSC: mesenchymal stem cell
qRT-PCR: Quantitative reverse-transcription polymerase chain reaction
SD: Sprague-Dawley
SMA: Smooth muscle actin
TD: Transplantation day
TGF: Transforming growth factor
TNF: Tumor necrosis factor
UC-MSC: umbilical cord-derived mesenchymal stem cell
VEGF: Vascular endothelial growth factor
