# Supplementary figures and images for: Therapeutic effect of human umbilical cord-derived mesenchymal stem cells on injured rat endometrium during its chronic phase
Source: Stem Cell Res Ther. 2018 Feb 13;9:36. doi: 10.1186/s13287-018-0777-5 (PMC5810045; doi:10.1186/s13287-018-0777-5)

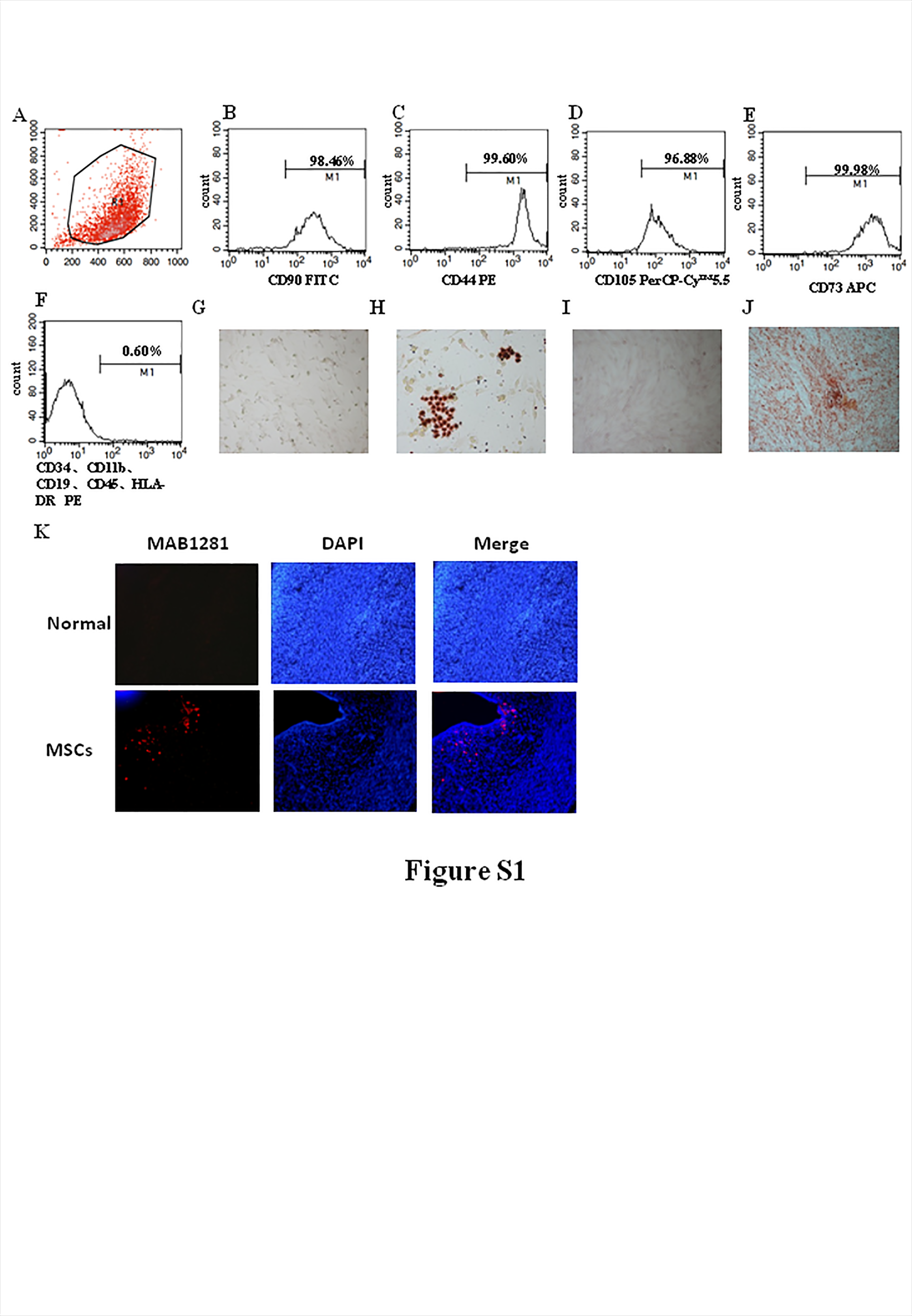

Supplement: Supplementary file 1 — Identification of stem cells. (A–F) Flow cytometry analysis of immune-markers in human UC-MSCs. (G) Normal human UC-MSCs was stained by Oil Red O (200×). (H) Adipogenesis was confirmed by Oil Red O staining to show intracellular lipid accumulation (200×). (I) Normal human UC-MSCs were stained by Alizarin Red (200×). (J) Osteogenesis was confirmed by Alizarin Red staining to show calcium deposition (200×). (K) MAB1281 expression in normal group and MSCs transplantation group at TD8 (200×). (TIF 6471 kb) [file 13287_2018_777_MOESM1_ESM.tif]

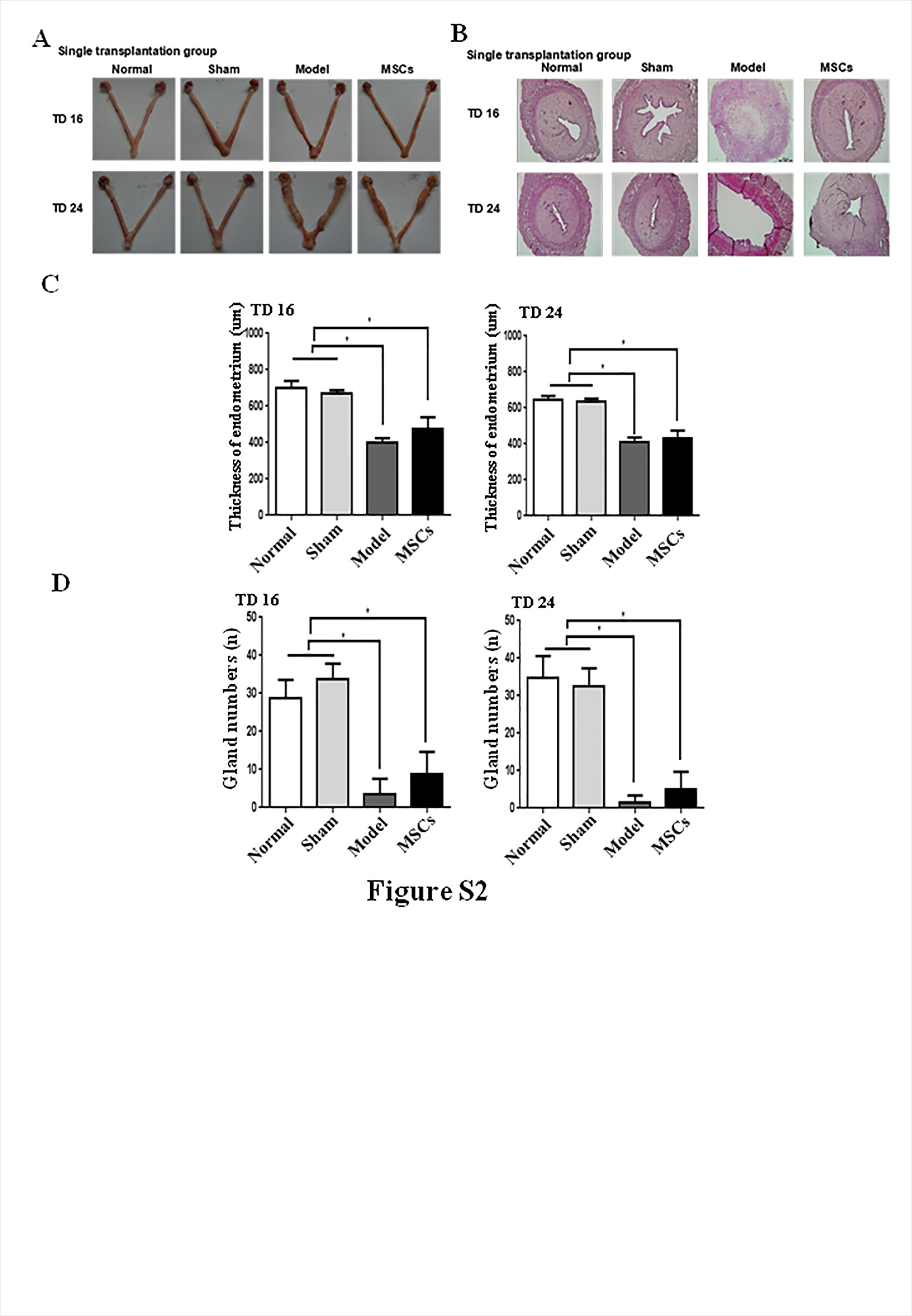

Supplement: Supplementary file 2 — Uterine morphological features and changes of single transplantation group on TD16 and TD24. (A) A uterus specimen. (B) H&E staining of rat uterine tissue (50×). (C) The endometrial thickness at TD16 and TD24. (D) The gland numbers at TD16 and TD24. *P < 0.05. (TIF 5634 kb) [file 13287_2018_777_MOESM2_ESM.tif]

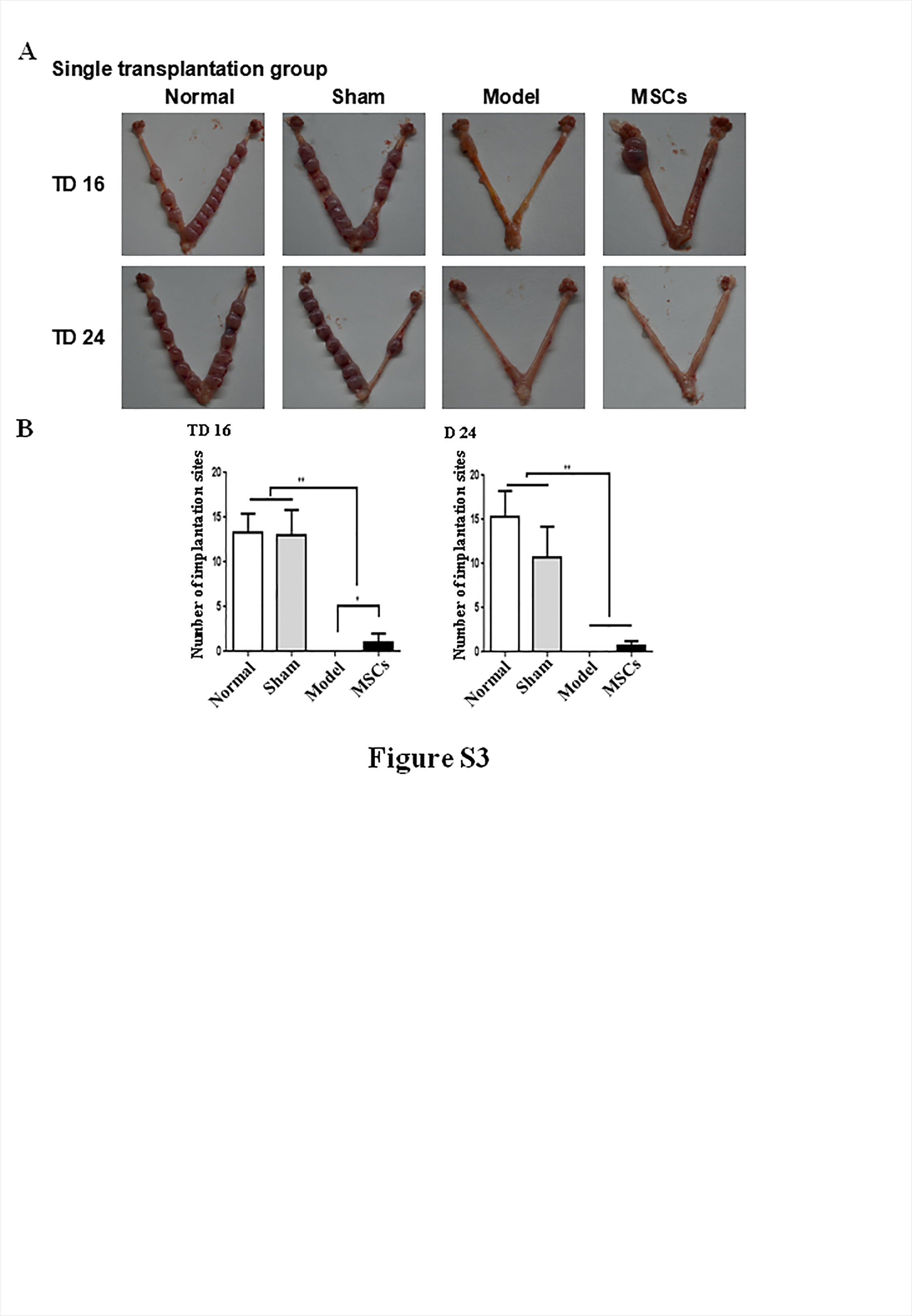

Supplement: Supplementary file 3 — Human UC-MSC transplantation restores receptive fertility of the single transplantation group at TD16 and TD24. (A) Embryos implanted in the uterus on TD16 and TD24. (B) The effect on rat pregnancy number by human UC-MSC transplantation at TD16 and TD24. *P < 0.05, **P < 0.01. (TIF 4634 kb) [file 13287_2018_777_MOESM3_ESM.tif]

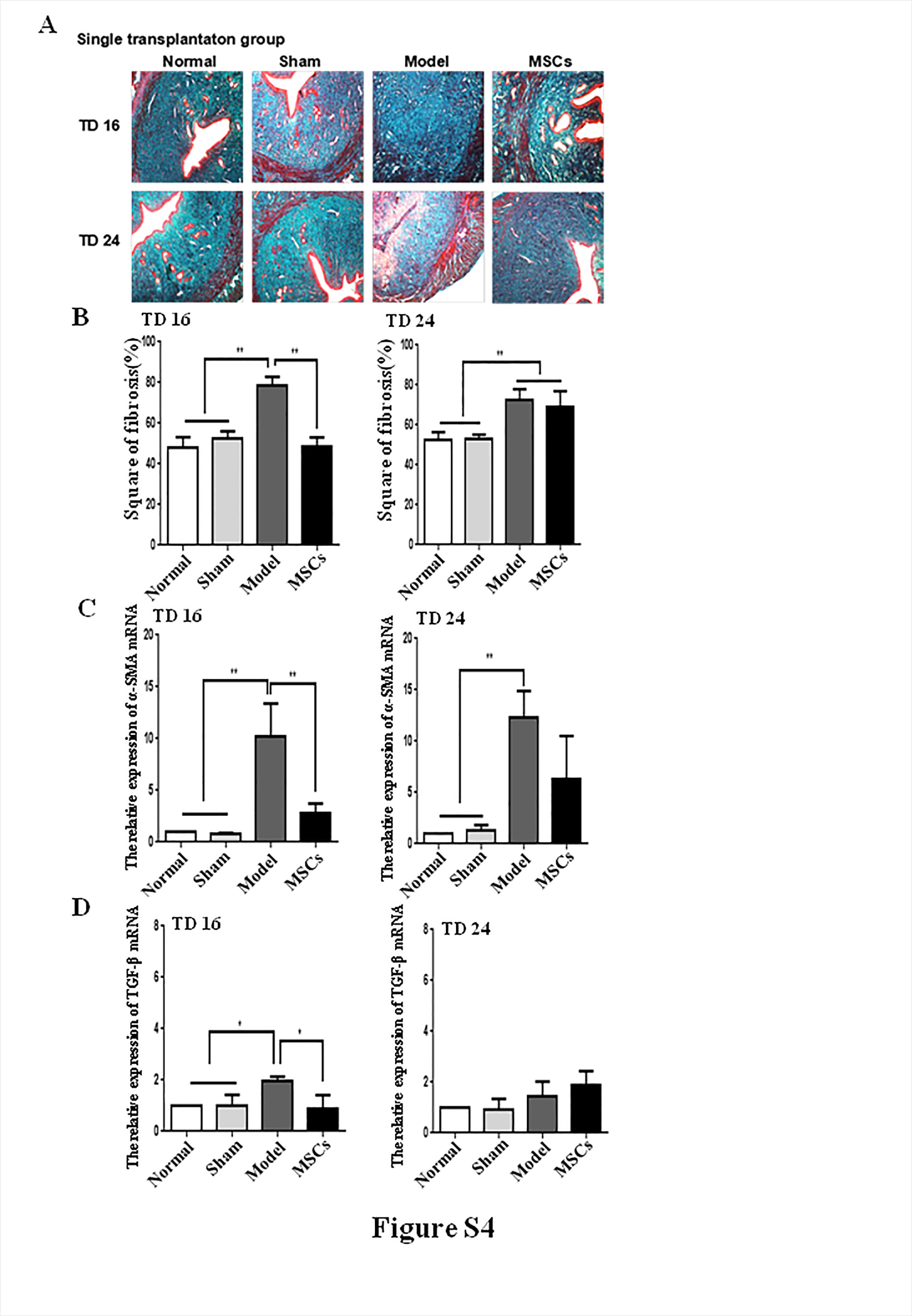

Supplement: Supplementary file 4 — Human UC-MSC transplantation relieves endometrial fibrosis of the single transplantation group at TD16 and TD24. (A) Masson’s trichrome staining of the single transplantation group at TD16 and TD24 (100×). (B) The fibrosis at TD16 and TD24. (C) α-SMA mRNA expression at TD16 and TD24 was detected by qRT-PCR. (D) TGF-β mRNA expression at TD16 and TD24 was detected by qRT-PCR. GAPDH serves as an internal reference for qRT-PCR. *P < 0.05, **P < 0.01. (TIF 6938 kb) [file 13287_2018_777_MOESM4_ESM.tif]

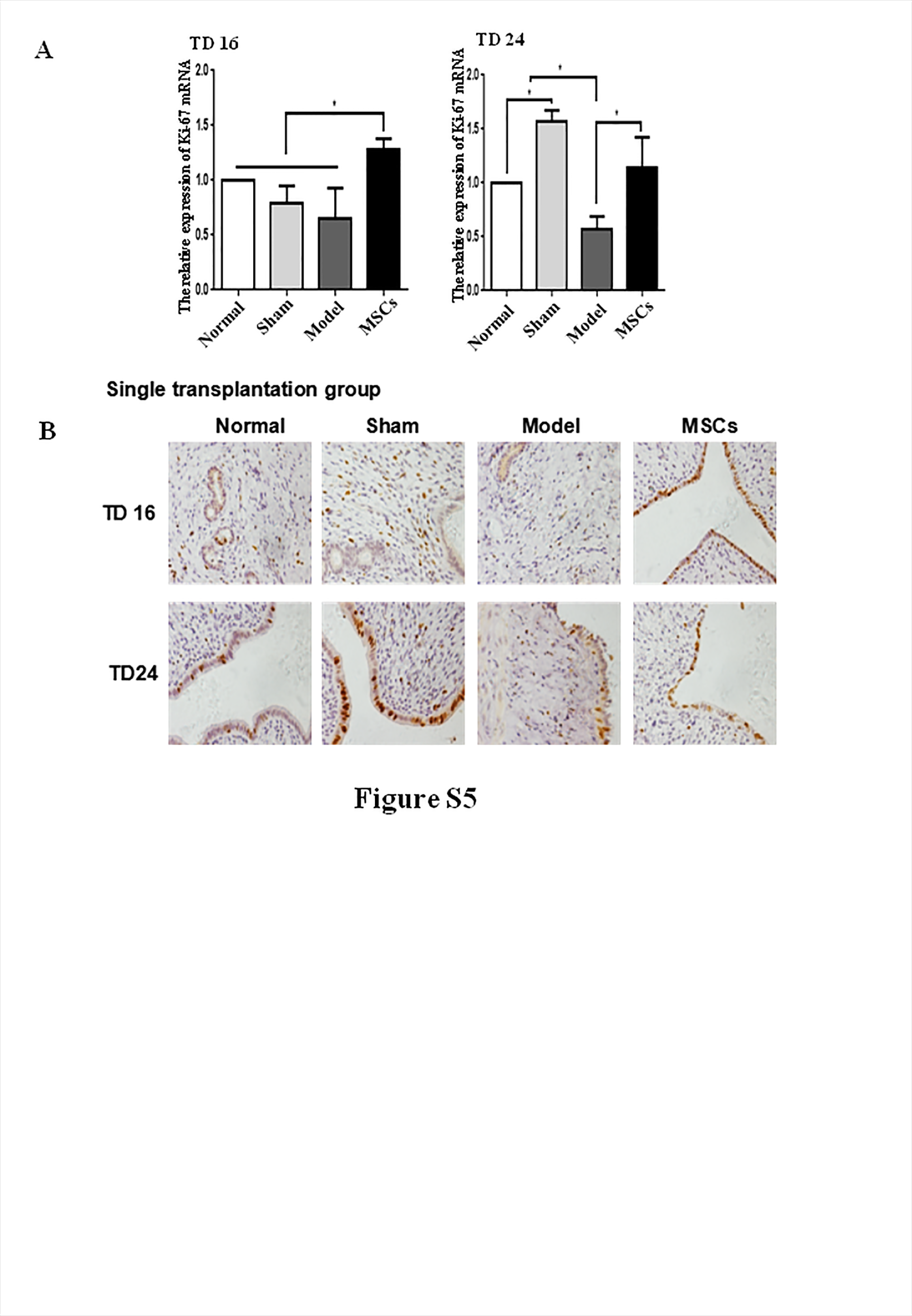

Supplement: Supplementary file 5 — Human UC-MSC transplantation promotes endothelial cell proliferation in the single transplantation group at TD16 and TD24. (A) Ki-67 mRNA expression at TD16 and TD24 was detected by qRT-PCR. GAPDH serves as an internal reference for qRT-PCR. *P < 0.05, **P < 0.01. (B) Ki-67 protein expression at TD16 and TD24 was detected by immunohistochemistry (400×). (TIF 6638 kb) [file 13287_2018_777_MOESM5_ESM.tif]

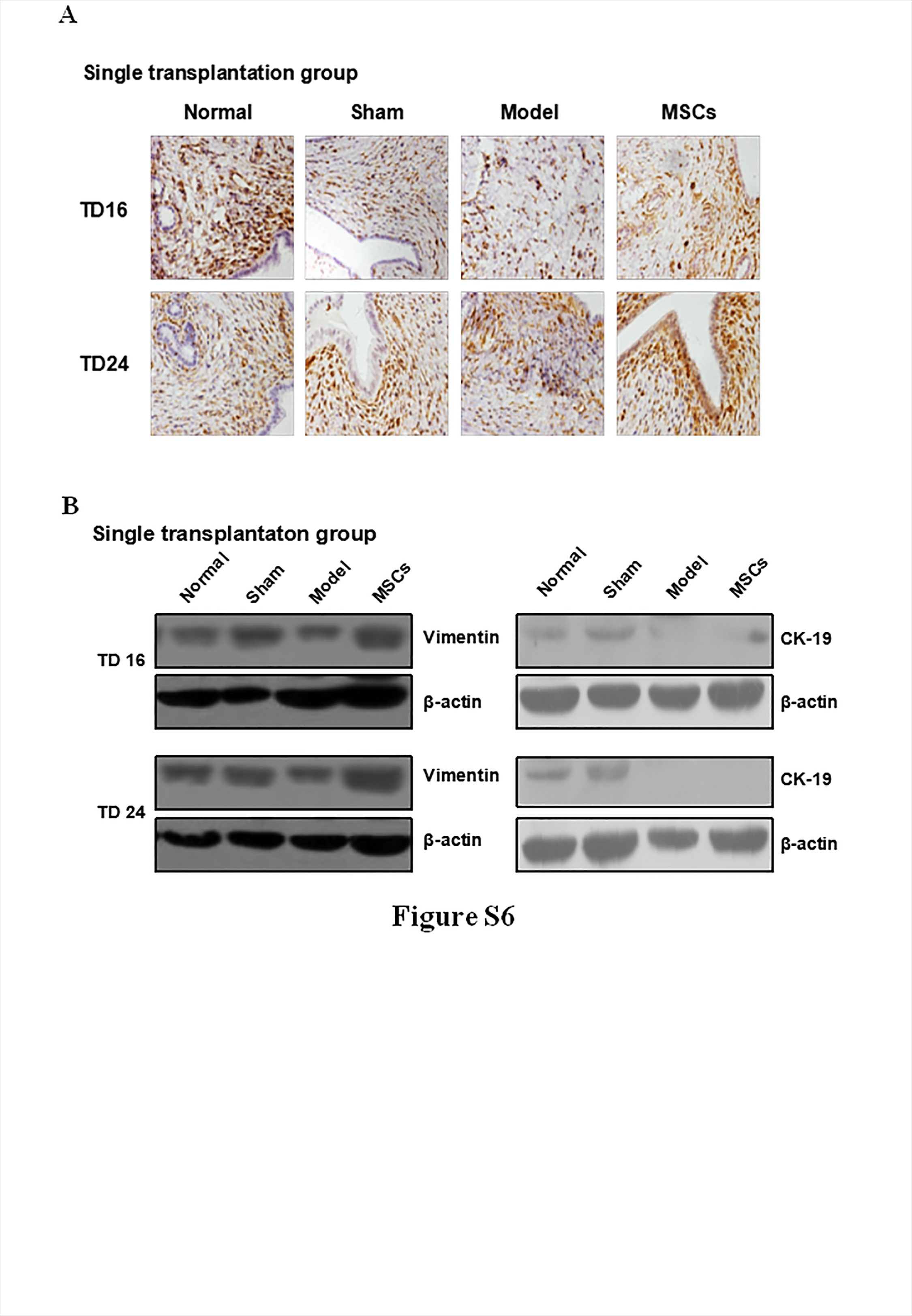

Supplement: Supplementary file 6 — Human UC-MSC transplantation promotes endometrial cell regeneration of the single transplantation group at TD16 and TD24. (A) Vimentin protein expression at TD16 and TD24 was detected by immunohistochemistry (400×). (B) Vimentin protein and CK-19 protein expression at TD16 and TD24 was detected by Western blot. (TIF 8220 kb) [file 13287_2018_777_MOESM6_ESM.tif]

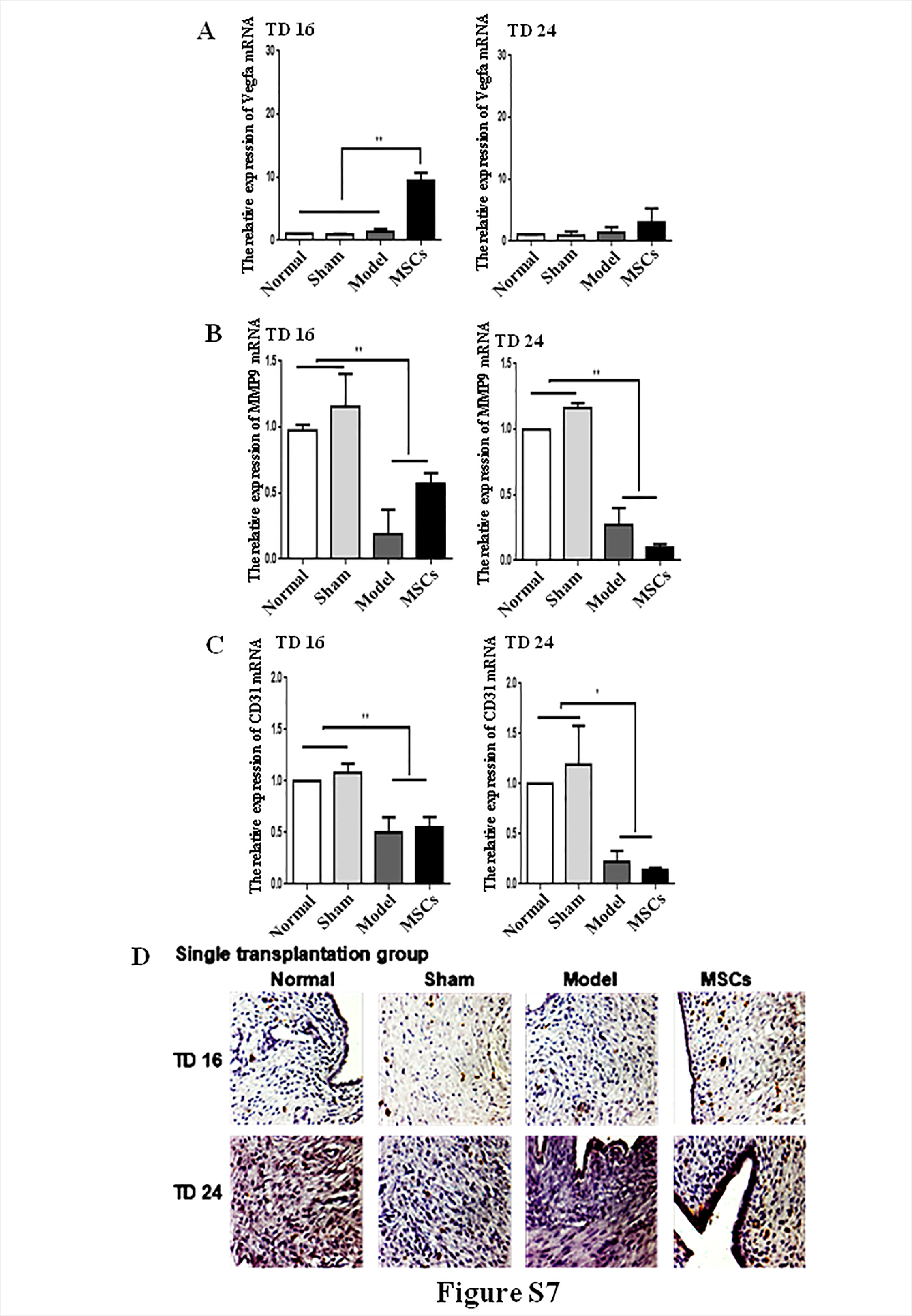

Supplement: Supplementary file 7 — UC-MSC transplantation promotes angiogenesis on TD16 and TD24 in the single transplantation group. (A) VEGFA mRNA expression at TD16 and TD24 was detected by qRT-PCR. (B) MMP9 mRNA expression at TD16 and 24 was detected by qRT-PCR. (C) CD31 mRNA expression at TD16 and TD24 was detected by qRT-PCR. GAPDH serves as an internal reference for qRT-PCR. *P < 0.05, **P < 0.01. (D) CD31 protein expression at TD16 and TD24 was detected by immunohistochemistry (400×). (TIF 8221 kb) [file 13287_2018_777_MOESM7_ESM.tif]
